# Examining the influence of a text message-based sleep and physical activity intervention among young adult smokers in the United States

**DOI:** 10.1186/s12889-015-2045-2

**Published:** 2015-07-16

**Authors:** A. Jordan Filion, Gerarda Darlington, Jean-Philippe Chaput, Michele Ybarra, Jess Haines

**Affiliations:** Department of Family Relations and Applied Nutrition, University of Guelph, 50 Stone Road East, Guelph, Ontario N1G 2 W1 Canada; Department of Mathematics and Statistics, University of Guelph, 50 Stone Road East, Guelph, Ontario N1G 2 W1 Canada; Healthy Active Living and Obesity Research Group, Children’s Hospital of Eastern Ontario Research Institute, 401 Smyth Road, Ottawa, Ontario K1H 8 L1 Canada; Center for Innovative Public Health Research, 555 North El Camino Real #A347, San Clemente, California 92672-6745 USA

**Keywords:** Mobile health, Young adults, Sleep, Physical activity, Smoking cessation, Text messaging

## Abstract

**Background:**

Sleep and physical activity are two health behaviors associated with improved smoking cessation outcomes. Text message-based interventions have previously been used to promote physical activity and smoking cessation; however, this type of intervention has not targeted sleep habits. This study examined the effectiveness of a text message-based active control intervention in improving sleep and physical activity habits among a U.S. national sample of young adult smokers participating in a smoking cessation intervention.

**Methods:**

This study was a secondary analysis of data from the Stop My Smoking USA randomized controlled trial. Baseline and 3-month follow-up data were collected from 116 young adult smokers (mean age 21.8 years, *SD* = 2.1) who were randomized at a 2:1 ratio to receive a 6-week text messaging program focused on either smoking cessation (*n* = 72), or improving sleep and physical activity (*n* = 44). Three main outcomes were assessed: 1) sleep quantity (on work/school nights, and non-work/non-school nights), 2) sleep quality, and 3) physical activity at follow-up. Multivariable linear regression analysis was used to quantify the differences in these outcomes between the groups. To identify possible effect modification by baseline sleep and physical activity, the sample was stratified by indicators defined for both of these variables.

**Results:**

At follow-up, sleep quantity and quality were similar for participants in the smoking cessation and sleep/activity groups when assessed among the total sample and those sleeping ≥6 hours/night at baseline. Among short sleepers (<6 hours/night at baseline), sleep quantity on work/school nights improved for those receiving sleep/activity messages compared to those receiving smoking cessation messages, after adjusting for covariates ($\widehat{\beta}$ =1.373, 95 % CI [0.262, 2.484]; *p* = 0.02). Physical activity at follow-up was similar for the two groups, when examined among the total sample and when stratified by baseline activity level.

**Conclusions:**

This study provides preliminary evidence that a text message-based intervention may be a promising approach for improving sleep quantity among young adult smokers who are short sleepers and interested in quitting smoking. Similar programs should be further explored as a novel approach for improving sleep habits among individuals with insufficient sleep.

**Trial registration:**

ClinicalTrials.gov NCT01516632

## Background

Health behavior patterns established during one’s young adult years have the potential to become life-long habits that can dictate one’s health status and risk for chronic disease [1, 2]. During this transitional period, individuals are more sensitive to environmental influences which, in addition to having the potential to motivate the adoption of positive health behaviors, can influence the adoption of poor health behaviors [3, 4]. Therefore, young adulthood can be viewed as a critical period for intervening on poor health behaviors, including smoking, insufficient sleep, and inactivity, before such behaviors become entrenched.

Sleep quantity and quality and physical activity are some of the many health behaviors that have been found to decline during young adulthood [5]. International sleep data collected in 2006 revealed that 21 % of young adults attending university were habitual short sleepers, defined as getting less than 7 hours of sleep on most nights [6]. Other population-based research suggests that a large proportion of young adults experience sleep-related problems, where it was found that only 11.5 % of undergraduate students in the sample were classified as having *good* sleep quality, assessed via self-report using the Sleep Quality Index [7, 8]. Examining trends in physical activity during young adulthood, several studies have reported dramatic declines in physical activity during this period [9–11]. One longitudinal study using data from the National Longitudinal Study of Adolescent Health found that of those participants who reported engaging in at least five weekly bouts of moderate-to-vigorous physical activity as adolescents, only 4.4 % maintained this level of physical activity as young adults [9]. These findings therefore support intervening among young adults to promote the development of healthful sleep and physical activity habits.

Cigarette smoking has been shown to negatively impact both sleep and physical activity [12–14]. It has been well-documented that smokers have lower subjectively measured sleep quality and experience more insomnia-like symptoms, compared with non-smokers [15–17]. One recent study examining both subjectively and objectively measured sleep among adult smokers found that, in addition to having lower subjectively measured sleep quality, adults who smoked had shorter sleep period time, longer sleep latency (i.e., took longer to fall asleep), higher rapid eye movement sleep density (indicating less restful sleep), and more sleep apneas and leg movements in sleep than non-smokers, all of which were objectively measured using polysomnography [13]. In addition, it has been established in the physical activity literature that cigarette smoking compromises cardiopulmonary function in the short-term, which could lead to reduced levels of physical activity [12, 14].

Improving sleep and physical activity habits are two of many recommended strategies to assist with smoking cessation [18]. Physical activity has been associated with improved weight control among those trying to quit smoking, and can also alleviate stress and assist with managing food and nicotine cravings and withdrawal symptoms experienced during the quitting process [18, 19]. Furthermore, physical activity can lead to improved sleep [20]. This, in turn, can provide individuals trying to quit smoking with the energy to cope with nicotine cravings and avoid the negative feelings that typically emerge as a result of being tired [21].

Using a text message-based intervention platform offers several other advantages compared with interventions involving face-to-face contact with participants, including great reach [22], cost-effectiveness [22], unobtrusiveness [22], the ability to collect data in real-time [22], and the ability to intervene anywhere, anytime [23]. Capitalizing on these advantages, text message-based behavior change interventions have recently been applied to a range of health behaviors, including smoking [24–28] and physical activity [29–35], with varying success. Text message-based smoking cessation interventions, in particular, have yielded promising results, with participation in the majority of such interventions being associated with increases in abstinence from cigarette smoking [24, 26–28].

To date, a text message-based intervention platform has not yet been used to improve sleep habits. In addition, all of the in-person interventions that have aimed to change sleep habits among young adults have been conducted exclusively among higher education students, so it remains unknown whether results from these interventions can be generalized to young adults outside of a tertiary education setting, particularly those who are engaging in, but interested in ceasing, health risk behaviors. Given the remarkable amount of time that young adults spend on mobile devices [36], implementing a text message-based sleep intervention may be a promising approach for improving young adults’ sleep habits.

Examining results from text message-based interventions targeting physical activity behavior, one intervention was associated with significant increases in physical activity [35], while another demonstrated no significant effect [34]. Prestwich and colleagues found that university students randomized to receive a 4-week intervention comprised of both creating a personalized exercise plan and receiving tailored text messages reminding them of that plan significantly increased their physical activity as compared to either approach alone and the control conditions [35]. Newton and colleagues found that adolescents with type 1 diabetes who were randomized to receive motivational text messages for 12 weeks while wearing a pedometer actually decreased their physical activity over the study period [34]. Studies examining the impact of physical activity interventions among groups engaging in other types of health risk behaviors, such as smoking, are lacking. Furthermore, no text message-based physical activity interventions designed exclusively for young adults who are trying to quit smoking exist in the literature.

This study addresses existing gaps in the literature by examining the effectiveness of a text message-based intervention on improving sleep and physical activity habits among a U.S. national sample of young adult smokers who were considering quitting smoking in the next 30 days. The current secondary analysis used data from the Stop My Smoking (SMS) USA randomized controlled trial, which is a 6-week text message-based smoking cessation program developed for 18–25 year old smokers in the United States [28]. Messages for an attention- matched control group were developed with a focus on improving one’s sleep and increasing one’s physical activity within the context of helping one quit smoking. The main results of the randomized controlled trial are reported elsewhere [28]. Reported here are the sleep and activity outcomes for the attention-matched control group (i.e., sleep/activity group) versus the smoking cessation group. This attention-matched control design is an efficient way to test the effect of interventions on multiple outcomes and allows the researcher to ascertain that any observed intervention effects are not a result of more attention being given to intervention group participants [37, 38].

It was hypothesized that participants randomized to the sleep/activity group would report higher levels of sleep quantity, sleep quality, and physical activity at follow-up as compared to participants randomized to the smoking cessation group. Additionally, it was hypothesized that the sleep/activity text message program would be more effective among those participants identified as short sleepers (i.e., those getting <6 hours of sleep per night) and/or inactive (i.e., those getting <150 minutes of physical activity per week) at baseline, based upon findings from an online sleep intervention study suggesting that only participants classified as *poor* sleepers at baseline experienced improvements in sleep quality at post-intervention [39].

## Methods

### Study design and recruitment

This study is a secondary analysis of the SMS USA randomized controlled trial [28]. Ethical approval for the SMS USA study, including the informed consent protocol, was obtained through Chesapeake Research Review Incorporated, and ethical approval for the current analysis of de-identified secondary data was obtained through the University of Guelph Institutional Review Board.

Young adults aged 18 to 25 years were recruited nationally through online advertisements on websites (primarily Craigslist) between May 2011 and August 2011. In addition to meeting the specified age criteria, eligible participants had to: be able to read and write in English, own a cell phone, be enrolled (or intend to enroll) in an unlimited text messaging plan, be familiar with how to send and receive text messages, smoke 24 cigarettes or more per week (i.e., at least 4 per day on at least 6 days per week), be seriously thinking about quitting smoking in the next 30 days, and agree to smoking cessation status verification by a significant other (i.e., family member or friend).

**Fig. 1:**
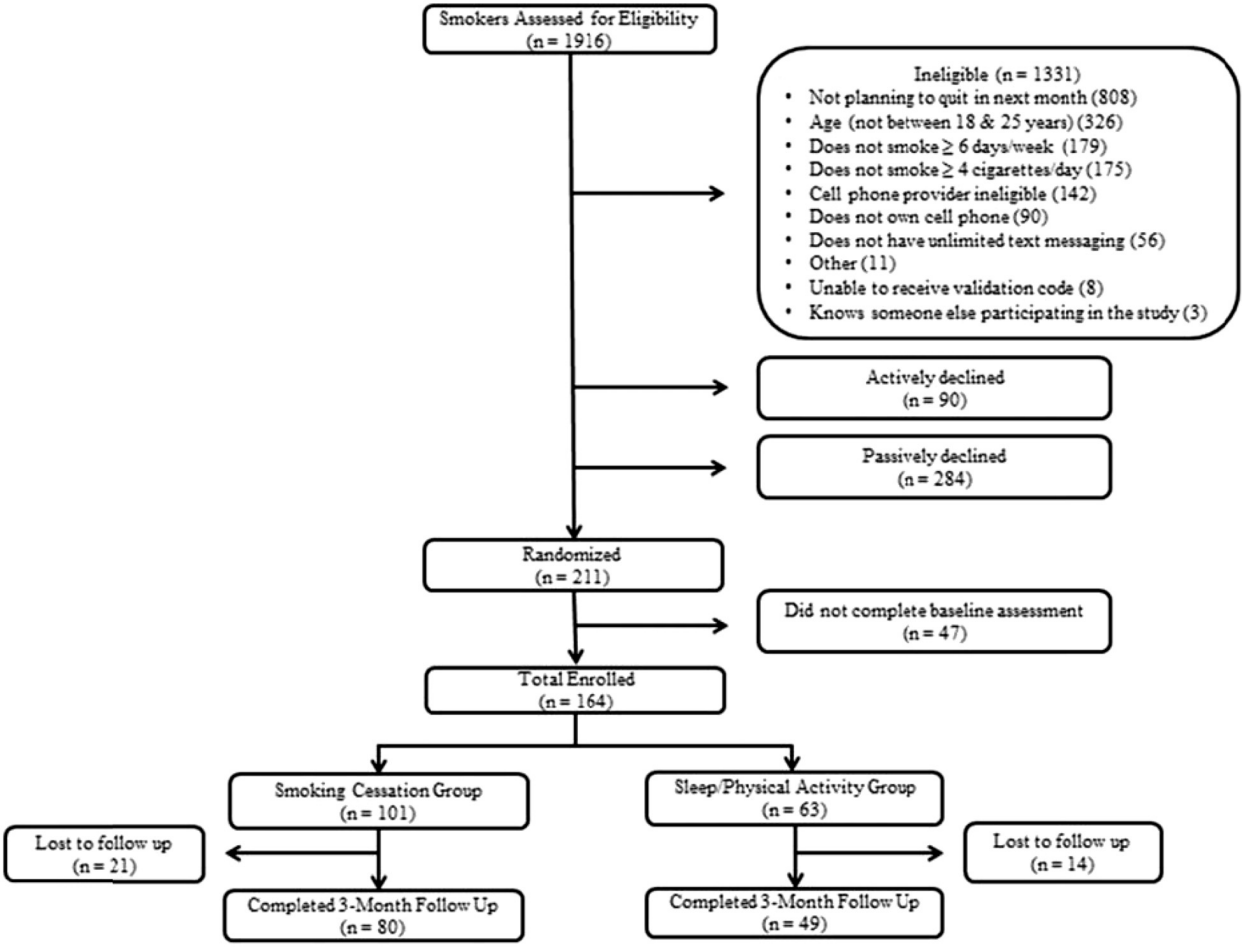
SMS USA study design and participant flow. Note: -Reasons for eligibility were not mutually exclusive, therefore, a participants could be ineligible for more than one reason. -Of the 80 participants who completed 12-week follow up measures, 4 were excluded from analysis because they were considered to be sleep outliers (reporting either 0 or 1 hour sleep/night on both work/school night & non-work/non-school nights)

Of the 1916 smokers assessed for eligibility, 211 met the study’s eligibility criteria and consented to participate, and 164 participants successfully enrolled in the study and completed baseline measures. Participants were either randomized to receive text messages that were tailored to their quit status and focused on smoking cessation (i.e., the “smoking cessation” group; *n* = 101), or aimed at improving sleep and physical activity habits within the context of how these behaviors may help them quit smoking (i.e., the “sleep/activity” group, *n* = 63). Participants who completed 3-month follow-up measurements (*n* = 129; 78.7 %) were included in the longitudinal analysis. The follow-up rate differs from previous reports due to missing data on the sleep and physical activity indicators at follow-up. Figure 1 describes the SMS USA study design and participant flow.

### Interventions

All participants (irrespective of study arm) were asked to identify a quit date that was at least 15 days, but no more than 30 days, from their registration date. Both smoking cessation and sleep/activity text messages began 14 days prior to one’s established quit date. Details regarding the development of the smoking cessation text message database are published elsewhere [40]. For the sleep/activity arm, text messages were initially developed by Ybarra and colleagues and then underwent expert review. Messages matched the number and flow of the smoking cessation group messages. There were a total of 144 text messages in the database for each study arm.

Smoking cessation group participants were exposed to a 6-week smoking cessation program with content that was tailored to where participants were in the quitting process (i.e., Day 1 to 14 or the Pre-Quit phase, Day 15 to 21 or the Early Quit phase, or Day 22 to 42 or the Late Quit phase). These participants received two weeks of Pre-Quit messages aimed at encouraging them to clarify reasons for quitting and to understand their smoking patterns, in addition to their tempting situations, triggers and urges. Both smoking cessation and sleep/activity group participants were allowed to define their “window” for receiving daily text messages (e.g., the number of hours over which text messages would be delivered to their mobile phone). Those participants who had the same window received text messages around the same time each day. Frequency of text message delivery ranged from as little as 1 message per day during the final week of the intervention, to 9 messages per day on Quit Day and Post-Quit Day 2.

Participants in the sleep/activity group received text messages at the same rate as the smoking cessation group in order to match the level of attention that the smoking cessation group was receiving; however, content of the text messages was aimed at improving participants’ sleep and physical activity habits within the context of how it would help the participant quit smoking (e.g., “*Sleeping and exercising go hand-in-hand when you’re trying to quit smoking. You have more energy, you sleep better, and it gives you the strength to quit*”, “*Regular exercise has a lot of benefits: Better sleep and relief from stress are just a couple. Remind yourself of YOUR reasons to make these life changes*”). All participants in the sleep/activity group received the same text messages, and both sleep- and activity-related messages were delivered on the same day.

### Measures

At baseline and follow-up, sleep quantity was assessed using two self-report items adapted from the Pittsburgh Sleep Quality Index (PSQI) [41]. Participants were asked to think about their sleep habits over the past 30 days and indicate how many hours they typically slept on work/school nights, and also on non-work/non-school nights (rounded to the nearest hour). Sleep quality was measured using 8 self-report items adapted from the PSQI, which all began with the stem “During the past month, how often have you…”, and included the following: (a) not been able to get to sleep within 30 minutes, (b) woken up in the middle of the night or early morning, (c) had to get up from sleeping to use the bathroom, (d) had trouble sleeping because you could not breathe comfortably, (e) had trouble sleeping because you were coughing or snoring loudly, (f) had trouble sleeping because you felt too cold or hot, (g) had bad dreams, and (h) had trouble sleeping because you were in pain. Response options for each item included: “not at all during the past month”, “less than once a week”, “once or twice a week”, “three or more times a week”, and “do not want to answer”. For the purposes of the current study, sleep quality scores were calculated for each participant by summing participants’ quantified responses to the 8 sleep quality items adapted from the PSQI. Cronbach’s alpha for the sleep quality scale used in the current study was 0.77 at baseline and 0.73 at 3-month follow-up, indicating acceptable internal consistency reliability [42]. Possible scores ranged from 8 to 32, with higher scores being indicative of poorer sleep quality, similar to the approach used by Urponen and colleagues [7]. None of the participants chose the response option “do not want to answer”.

Physical activity was measured using two self-report items adapted from the National Health Interview Survey [43]. Participants were asked to think about their physical activity habits over the past 30 days and indicate in separate questions how many days per week they engaged in vigorous leisure-time physical activity (i.e., activities causing heavy sweating or large increases in breathing or heart rate) and light-to-moderate leisure-time physical activity (i.e., activities causing only light sweating or a slight to moderate increase in breathing or heart rate) for at least ten minutes. Participants were also asked to indicate the approximate length of time they performed each type of physical activity. The number of hours of physical activity per week was calculated for both types of physical activity at baseline and follow-up by multiplying the self-reported frequency of physical activity by the duration, and then adding these figures together to compile a total physical activity score [44].

### Statistical analysis

All statistical analyses were conducted using version 21 of SPSS Statistics for Windows (PASW, IBM, New York, USA). Separate linear regression analyses were used to examine the impact of the sleep/activity messages on 3-month follow-up levels of: 1) sleep quantity (i.e., the average number of hours slept on work/school nights, and non-work/non-school nights); 2) sleep quality (e.g., sleeping through the night); and 3) physical activity (i.e., the number of days per week one engaged in leisure-time physical activity). To determine whether the sleep/activity content had differential effects for short sleepers versus adequate sleepers and inactive participants versus active participants, intervention effects were examined by baseline sleep category, i.e., short sleepers (<6 hours/night; *n* = 34) versus those who slept ≥6 hours/night (*n* = 82), and baseline physical activity level, i.e., inactive participants (<150 minutes/week activity recommendation from the 2008 Physical Activity Guidelines for Americans [45] (*n* = 43)) versus active participants getting ≥150 minutes/week (*n* = 73). In the first model, only baseline habits were controlled for. In the fully adjusted models, corresponding baseline habits were controlled for, in addition to quit status at follow-up, daily text messaging usage (i.e., number of text messages sent on a typical day), sex, educational attainment (i.e., currently enrolled in post-secondary education/post-secondary completer or high school or less/post-secondary non-completer), race (i.e., Caucasian or other), change in physical activity (in the sleep models), and change in average sleep quantity (in the physical activity models).

## Results

### Sample

Participants whose responses to the items assessing sleep quantity or physical activity were greater than three standard deviations from the mean were considered to be outliers. There were no substantive differences in demographics among the outliers excluded from the analyses (*n* = 13) and participants retained in the final analytical sample (*n* = 116; data not shown).

Table 1 presents a summary of participants’ demographic/baseline characteristics by study arm. There were no differences between participants in the sleep/activity group and smoking cessation group with respect to the demographic/baseline variables examined, with the exception of baseline sleep quantity on work/school nights ($\overline{X} = 5.82$ hours in the sleep/activity group versus 6.42 hours in the smoking cessation group; *p* = 0.03) and non-work/non-school nights ($\overline{X} = 7.50$ hours in the sleep/activity group versus 8.32 hours in the smoking cessation group; *p* = 0.04).

**Table 1:** Demographic/baseline characteristics of participants in the SMS USA study (overall and by intervention assignment)

	Overall (n = 116)	Smoking cessation group (n = 72)	Sleep/activity group (n = 44)	*P*-value
Demographic/baseline characteristics				
		N (%)		
Sex				
Male	61 (52.6)	40 (55.6)	21 (47.7)	0.45
Female	55 (47.4)	32 (44.4)	23 (52.3)	
Race				
White or Caucasian	78 (67.2)	47 (65.3)	31 (70.5)	0.68
Other	38 (32.8)	25 (34.7)	13 (29.5)	
Educational attainment				
High school or less/post-sec non-completer	24 (20.7)	13 (18.1)	11 (25.0)	0.48
Currently enrolled in post-sec/post-sec completer	92 (79.3)	59 (81.9)	33 (75.0)	
	Median (25 %, 75 % quartile)			
Daily text messaging usage	50 (20, 100)	50 (20, 100)	43.5 (18.5, 100)	0.46
		Mean (SD)		
Sleep quantity on work/school nights (hours)	6.19 (1.45)	6.42 (1.44)	5.82 (1.40)	0.03*
Sleep quantity on non-work/non-school nights (hours)	8.01 (2.11)	8.32 (2.05)	7.50 (2.14)	0.04*
Sleep quality score	17.20 (5.21)	17.00 (5.50)	17.52 (4.74)	0.60
Physical activity (hours/week)	5.70 (6.58)	6.38 (7.41)	4.59 (4.82)	0.16

*Significant at *p* < 0.05

### Linear regression results: sleep quantity and quality

None of the follow-up sleep measures differed significantly between the sleep/activity and smoking cessation groups when all participants were examined together (Table 2), both in the partly adjusted and fully adjusted models.

**Table 2:** Relative difference in sleep indicators between groups at 3-month follow-up

Sleep measure	Baseline mean (SD)	12-week follow-up mean (SD)	Unadjusted difference β (95 % CI)^a^	P-value	Adjusted difference β (95 % CI)^b^	*P*-value
Sleep quantity on work/school nights (hours/night)						
Sleep/activity group	5.82 (1.40)	6.34 (1.41)	0.306 (−0.155, 0.766)	0.19	0.335 (−0.134, 0.805)	0.16
Smoking cessation group	6.42 (1.44)	6.39 (1.48)				
Sleep quantity on non-work/non-school nights (hours/night)						
Sleep/activity group	7.50 (2.14)	7.93 (2.05)	0.060 (−0.649, 0.769)	0.87	−0.023 (−0.724, 0.678)	0.95
Smoking cessation group	8.32 (2.05)	8.18 (1.95)				
Sleep quality score^c^						
Sleep/activity group	17.52 (4.74)	15.82 (4.61)	0.074 (−1.529, 1.676)	0.93	0.078 (−1.548, 1.705)	0.92
Smoking cessation group	17.00 (5.50)	15.53 (4.80)				

*SD* Standard deviation, *CI* Confidence interval

*n* = 116

^a^Adjusted for baseline sleep

^b^Adjusted for baseline sleep, change in physical activity, smoking status at follow-up, daily text messaging usage, sex, educational attainment, and race

^c^Sleep quality was assessed using 8 self-report items adapted from the PSQI; scores were the sum of participants' quantified responses for each item, with higher scores being indicative of poorer sleep quality (possible scores ranged from 8 to 32)

Among short sleepers at 3-month follow-up, sleep quantity on work/school nights increased by a mean of 31 minutes/night among participants in the sleep/activity arm and decreased by 2 minutes/night among participants in the smoking cessation arm, with a non-significant unadjusted difference of 1.04 hours (62 minutes; 95 % CI (−0.095, 2.172; Table 3)). After adjusting for covariates (i.e., change in physical activity, smoking status at follow-up, daily text messaging usage, sex, educational attainment, and race), the difference increased to 1.37 hours (82 minutes; 95 % CI (0.262, 2.484)) and was statistically significant. Sleep quantity on non-work/non-school nights and sleep quality were not statistically different among short sleepers in the sleep/activity and smoking cessation groups, however. No intervention effects were found for any of the sleep outcomes among adequate sleepers (*n* = 82; Table 4).

**Table 3:** Relative difference in sleep indicators between groups at 3-month follow-up for short sleepers (<6 hours/night)

Sleep measure	Baseline mean (SD)	12-week follow-up mean (SD)	Unadjusted difference β (95 % CI)^a^	*P*-value	Adjusted difference β (95 % CI)^b^	*P*-value
Sleep quantity on work/school nights (hours/night)						
Sleep/activity group	4.27 (0.70)	5.87 (2.00)	1.039 (−0.095, 2.172)	0.07	1.373 (0.262, 2.484)*	0.02**
Smoking cessation group	4.53 (0.84)	5.05 (1.43)				
Sleep quantity on non-work/non-school nights (hours/night)						
Sleep/activity group	5.87 (2.23)	6.93 (2.02)	0.175 (−1.464, 1.815)	0.83	−0.332 (−2.135, 1.470)	0.71
Smoking cessation group	7.74 (2.54)	7.16 (2.32)				
Sleep quality score^c^						
Sleep/activity group	19.33 (4.72)	17.33 (5.25)	−1.468 (−4.570, 1.633)	0.34	−1.620 (−4.821, 1.581)	0.31
Smoking cessation group	18.89 (5.13)	18.58 (4.81)				

*SD* Standard deviation, *CI* Confidence interval

*n* = 34

^a^Adjusted for baseline sleep

^b^Adjusted for baseline sleep, change in physical activity, smoking status at follow-up, daily text messaging usage, sex, educational attainment, and race

^c^Sleep quality was assessed using 8 self-report items adapted from the PSQI; scores were the sum of participants' quantified responses for each item, with higher scores being indicative of poorer sleep quality (possible scores ranged from 8 to 32)

*95 % CI does not contain 0

**Significant at *p* < 0.05

**Table 4:** Relative difference in sleep indicators between groups at 3-month follow-up for participants sleeping ≥6 hours/night

Sleep measure	Baseline mean (SD)	12-week follow-up mean (SD)	Unadjusted difference β (95 % CI)^a^	*P*-value	Adjusted difference β (95 % CI)^b^	*P*-value
Sleep quantity on work/school nights (hours/night)						
Sleep/activity group	6.62 (0.90)	6.59 (0.95)	−0.006 (−0.467, 0.456)	0.98	0.001 (−0.481, 0.483)	1.00
Smoking cessation group	7.09 (0.90)	6.87 (1.18)				
Sleep quantity on non-work/non-school nights (hours/night)						
Sleep/activity group	8.34 (1.54)	8.45 (1.90)	−0.027 (−0.780, 0.726)	0.94	0.027 (−0.719, 0.774)	0.94
Smoking cessation group	8.53 (1.83)	8.55 (1.68)				
Sleep quality score^c^						
Sleep/activity group	16.59 (4.55)	15.03 (4.12)	0.517 (−1.305, 2.339)	0.57	0.468 (−1.395, 2.332)	0.62
Smoking cessation group	16.32 (5.52)	14.43 (4.34)				

*SD* Standard deviation, *CI* Confidence interval

*n* = 82

^a^Adjusted for baseline sleep

^b^Adjusted for baseline sleep, change in physical activity, smoking status at follow-up, daily text messaging usage, sex, educational attainment, and race

^c^Sleep quality was assessed using 8 self-report items adapted from the PSQI; scores were the sum of participants' quantified responses for each item, with higher scores being indicative of poorer sleep quality (possible scores ranged from 8 to 32)

### Linear regression results: physical activity

When examining differences among the total sample, results indicated that there was no significant difference in physical activity between the sleep/activity group and smoking cessation group at follow-up (Table 5). This was observed in both the partly adjusted and fully adjusted models. When the sample was stratified by level of baseline physical activity, no evidence of effect modification was found (results not shown).

**Table 5:** Relative difference in physical activity between groups at 3-month follow-up

	Baseline mean (SD)	12-week follow-up mean (SD)	Unadjusted difference β (95 % CI)^a^	*P*-value	Adjusted difference β (95 % CI)^b^	*P*-value
Physical activity (hours/week)						
Sleep/activity group	4.59 (4.82)	6.32 (7.88)	−2.405 (−6.107, 1.296)	0.20	−1.929 (−5.683, 1.825)	0.31
Smoking cessation group	6.38 (7.41)	9.26 (10.85)				

*SD* Standard deviation, *CI* Confidence interval

*n* = 116

^a^Adjusted for baseline physical activity

^b^Adjusted for baseline physical activity, change in average sleep quantity, smoking status at follow-up, daily text messaging usage, sex, educational attainment, and race

## Discussion

Among young adult smokers 18 to 25 years of age who were thinking seriously about quitting and were recruited online across the United States, text messages that promoted improved sleep and increased physical activity to help one quit smoking were not associated with increases in either outcome over time. Among short sleepers (i.e., individuals getting <6 hours of sleep on work/school nights at baseline), young adult smokers in the sleep/activity intervention arm increased their sleep quantity on work/school nights at follow-up, as compared to those receiving the smoking cessation intervention. Baseline physical activity level was *not* found to influence the effect of the sleep/activity intervention on physical activity.

In the current study, among short sleepers who received the sleep/activity intervention, the mean increase in sleep quantity on work/school nights was 82 minutes. This magnitude of effect is higher than what has been reported in previous studies that have successfully improved sleep quantity among young adults. Prestwich and colleagues found that receiving a combination of sleep health education and keeping sleep logs resulted in a mean improvement of approximately 54 minutes of sleep per night among participants in their sample of college students [46]. In addition, Ball and Bax found that receiving a self-care intervention focused on improving sleep hygiene habits was associated with a significantly lower reduction in sleep quantity among first year medical school students (mean reduction was 10 minutes per night, compared to a 46-minute reduction observed among participants receiving a self-awareness intervention in which they received feedback on their Epworth Sleepiness Scale scores) [47]. It is possible that the frequency of the current intervention (i.e., multiple daily text messages versus a single educational session) may have contributed to the higher magnitude of change in sleep quantity observed in this study.

Results from studies designed to improve sleep in the general population have yielded mixed results, with the slight majority of studies finding statistically significant effects [39, 46–49], and other studies finding no effects [50], or small, non-significant effects [51–53]. There are several reasons that may explain why this study did not improve sleep habits in the sample as a whole. Perhaps most importantly, this group did not express any interest in changing sleep or physical activity behavior. They were recruited based upon their interest in quitting smoking. It seems likely then that these participants were at least less if not completely unmotivated to change these two behaviors as compared to participants in other sleep and physical activity interventions. Second, the content delivered via text message in the present study was very brief in comparison to sleep interventions that have been delivered face-to-face or via printed materials, which limited the amount of information that could be delivered at once (i.e., text messages primarily consisted of simple tips). Furthermore, the content of the text messages sent to participants in the sleep/activity group was divided between two health behaviors. It is possible that if one behavior had been the focus, a stronger effect may have been observed. Moreover, the sole reliance on text messages may have dampened the potential impact. For example, other text messaging interventions, including the cessation arm in the current study, have integrated other elements, including websites or email [26, 28, 29, 33], in-person visits or individual/group training on health behaviors [30, 32, 54, 55], printed materials [30, 31], and phone calls [31]. It is possible that multi-modal interventions are better able to affect behavior change, although a recent meta-analysis suggests that additional intervention components are not associated with increased likelihood of behavior change [56]. Additionally, although this study incorporated both educational messages (i.e., in the form of tips and strategies for improving sleep habits) and motivational/supportive messages, text message content was not tailored to participants’ baseline habits. It is therefore plausible that some participants, especially those with good sleep habits at baseline, may have found that the content was not relevant or did not apply to them if they were already performing the suggested behaviors. This is consistent with our finding that the sleep/activity intervention was more effective for participants who were classified as short sleepers at baseline. Finally, there was no way of knowing in the current study whether participants read the texts that were sent to their mobile phones. Process data reported in Ybarra and colleagues’ study, however, indicated that 20 % of sleep/activity participants reported *somewhat agreeing* or *strongly agreeing* when asked if they stopped reading the text messages by the end of the program [28]. This finding suggests that it is possible that significant behavior change did not occur because a sizeable proportion of the sample did not receive the full dose of the sleep/activity intervention.

Results showed that the sleep/activity intervention was not effective at improving physical activity among participants in the sleep/activity group. These results are similar to findings from a text message-based intervention to improve physical activity among adolescents with type 1 diabetes, where no improvements were observed for mean daily step count at post-intervention [34]. Similar to the current study, Newton and colleagues’ intervention was solely text message-based and relied on self-reported physical activity data, which could have led to an overestimation of physical activity. Conversely, results from another study using text messaging to improve physical activity among university students found a meaningful increase in physical activity among intervention group participants receiving a series of tailored text message reminders about their personal physical activity plans [35]. In contrast to the current study, both the content and frequency of the text messages in Prestwich and colleagues’ study was chosen by the participant prior to beginning the intervention and could be changed at any time throughout the 4-week program, thereby tailoring the intervention to the specific needs of each participant. In a recent meta-analysis on the efficacy of text message-based interventions for health promotion, use of message tailoring and personalization were found to be significantly associated with greater intervention efficacy [56].

Comparing the present findings for physical activity to what has been found in the literature, there are several potential explanations for why this study was not able to replicate the significant results found in Prestwich and colleagues’ intervention study. In addition to the possibilities noted above, it is plausible that physical activity may be harder to affect among individuals with pre-existing conditions or who are performing certain health behaviors. Unlike the healthy participants in Prestwich and colleagues’ study, in both our study which included smokers, and Newton and colleagues’ study which included individuals with diabetes, physical activity was not affected. It can be quite difficult for individuals who smoke to engage in more vigorous types of physical activity due to the shortness of breath associated with long-term cigarette smoking. It may be that sequencing smoking cessation first and physical activity subsequently is a more effective health promotion strategy.

Some key limitations should be kept in mind when interpreting results from the current study. First, data were from a text message-based smoking cessation intervention. As such, assessment of sleep and physical activity was not optimal, which could have introduced bias towards the null hypothesis. More comprehensive and observer-reported measures (e.g., actigraphy) would have been preferred. That said, self-report is a key indicator for the majority of behavior change studies, and often is found to produce a similar magnitude of effect when compared with biological indicators [57, 58]. Second, the focus on smokers and the recruitment method (i.e., online advertisements such as Craigslist) used in the current study may have limited the generalizability of study findings to the broader population of American young adults [28]. Third, with a modest sample size of 116 participants, power would not be large. It is possible that with a larger sample size, the two study arms may have differed with respect to the observed effect sizes. Lastly, participants were asked to think about their behavior over the past month. Therefore, in addition to the potential for social desirability bias to affect participants’ responses, the accuracy of participants’ responses may also have been affected by recall error when having to estimate how they slept or exercised, on average, over the past 30 days.

## Conclusions

This study provides preliminary evidence that a text message-based intervention may be a promising approach for improving sleep quantity among young adult smokers who are thinking about quitting smoking, especially short sleepers. Findings suggest that this mode of intervention delivery should be further explored as a novel approach to improving sleep habits among young adults. Future research should focus on targeting short sleepers and address the root causes of their short sleep using a tailored approach to maximize the relevance of information being delivered. Additional research incorporating more objective measures of sleep (i.e., actigraphy) and physical activity (i.e., accelerometry), and with participants who are interested in improving their sleep and physical activity, is needed before any definitive conclusions can be made regarding whether a text message-based intervention is an effective way to improve sleep and physical activity habits among young adults.

## Abbreviations

PSQI: Pittsburgh sleep quality index
SMS USA: Stop My Smoking USA
